# Genotoxicity Associated with Residual Monomers in Restorative Dentistry: A Systematic Review

**DOI:** 10.3290/j.ohpd.b2081469

**Published:** 2021-09-30

**Authors:** Mario José Romo-Huerta, Andréa del Refugio Cervantes-Urenda, José Velasco-Neri, Olivia Torres-Bugarín, Andréa Dolores Correia Miranda Valdivia

**Affiliations:** a Postgraduate Student, Department of Prosthodontics, Faculty of Dentistry, Autonomous University of Guadalajara, Guadalajara, Jalisco, México. Collected and interpreted the data, manuscript preparation, manuscript editing.; b Postgraduate Student, Department of Prosthodontics, Faculty of Dentistry, Autonomous University of Guadalajara, Guadalajara, Jalisco, México. Collected and interpreted the data, manuscript preparation.; c Professor, Department of Prosthodontics, Faculty of Dentistry, Autonomous University of Guadalajara, Guadalajara, Jalisco, México. Methodological design, proofread the manuscript.; d Researcher Professor, Genotoxic Research Laboratory, Department of Internal Medicine II, Autonomous University of Guadalajara, Guadalajara, Jalisco, México. Contributed to author tables, scientific advisor, proofread the manuscript.; e Researcher Professor, Department of Prosthodontics, Faculty of Dentistry, Autonomous University of Guadalajara, Guadalajara, Jalisco, México. Methodological design, scientific advisor, contributed substantially to manuscript, proofread the manuscript.

**Keywords:** dental resin, genotoxicity, methacrylates, residual monomers

## Abstract

**Purpose:**

Incomplete polymerisation processes produce several leachable substances. The aim of this work was to review, through existing research and published literature, the genotoxic effect of residual monomers of polymers used in restorative dentistry.

**Materials and Methods:**

The selection of published studies was performed on six databases from January 2000 to June 2020. The keywords used were: ‘genotoxicity’ or ‘DNA damage’ and ‘dental resin’ or ‘methacrylates’ or ‘residual monomers’. The selection was carried out according to the parameters and guidelines of the Preferred Reporting Items for Systematic Review and Metanalyses (PRISMA) and was based on patient, intervention, comparison, and outcome (PICO). The inclusion criteria were: in vitro and in vivo studies published in English that evaluated genotoxicity for residual monomers leached from polymers related to restorative dentistry. Case reports and review articles were excluded.

**Results:**

Twenty-seven studies met the eligibility criteria. Two categories were constructed based on the experimental design, in vivo and in vitro reports. For the in vitro research, two main methods of assessing DNA damage were reported in selected studies: micronucleus (MN) counting and alkaline comet assay. For in vivo reports, the main method for assessing genotoxic damage was MN counting.

**Conclusion:**

From the electronic search, structured data extraction, and analysis by different independent reviewers, results from the present systematic review allow us to conclude that DNA damage is induced by monomers/co-monomers (triethylene glycol dimethacrylate, bisphenol-A-glycidyl methacrylate, urethane dimethacrylate, and 2-hydroxyethyl methacrylate) that are used in restorative dentistry. This systematic review highlights the need for more research on the use of monomers/co-monomers to properly assess clinical biocompatibility.

Dentistry uses various polymer materials based on methacrylates. The matrix of these dental materials contains highly viscous major monomers such as bisphenol-A-glycidyl methacrylate (bis-GMA) or urethane dimethacrylate (UDMA), as well as dilutive monomers such as 2-hydroxyethyl methacrylate (HEMA) or the comonomer triethylene glycol dimethacrylate (TEG-DMA).^9^ The curing of restorative materials and adhesives is initiated chemically by mixing two components or by light. In both cases, polymerisation is incomplete, so varying amounts of free and unreacted monomers remain in the polymerised resin.^4,12,20^ The initial release of free monomers may occur during monomer–polymer conversion, and the long-term release of leachable substances is caused by erosion and degradation over time. Degradation of composites and polymers in the oral environment is caused by thermal changes, the components of saliva, chewing forces, chemical dietary changes, and oral microorganisms.^14,22,38^

Furthermore, monomers/co-monomers have the potential to increase the levels of reactive oxygen species (ROS). ROS are known mediators of signaling cascades, but elevated levels of ROS can disrupt the cellular redox balance, resulting in oxidative DNA damage and apoptosis in mammalian cells. Along this line, the ROS attack on DNA might induce adverse toxic effects such as mutagenicity and genotoxicity in the affected cells and organisms.^13,20,37^ A genotoxic agent is one that induces point mutations, deletions, insertions, gene amplification, chromosomal rearrangements, or numerical chromosomal changes. In the context of short-term tests for mutagenicity and genotoxicity, tests are designed to detect one or more types of genetic alterations. Since such biological properties result directly or indirectly from DNA damage, no single assay, no matter how extensive the protocol, can detect all genotoxic chemicals. Therefore, it is generally accepted that several tests must be conducted to evaluate whether a chemical is genotoxic or not, and often the weight-of-evidence approach must be taken to evaluate the results.^33^

Genomic damage is probably the most important fundamental cause of developmental and degenerative diseases. It is also well established that genomic damage is produced by environmental exposure to genotoxins, medical procedures (e.g. radiation and chemicals), micronutrient deficiency (e.g. folate), lifestyle factors (e.g. alcohol, smoking, drugs, and stress), and genetic factors such as inherited defects in DNA metabolism and/or repair.^7,16,43^ Several studies have investigated and identified the cytotoxicity and genotoxicity of some of these methacrylates during the last two decades.^17,19,32,34,35^ Resin monomers such as TEG-DMA or HEMA induced cytotoxicity via apoptosis in various cell types, including pulp and gingiva cells; genotoxic or mutagenic effects caused by monomers were reported as well.^18^ However, some studies report non-significant DNA damage and nuclear changes with the use of more dilute concentrations, which would resemble clinical conditions.^8,42^

This review evaluated, through existing research and publish literature, the genotoxic effect of residual monomers of polymers used in restorative dentistry.

## Materials and Methods

This study was carried out according to the parameters and guidelines of the Preferred Reporting Items for Systematic Review and Metanalyses (PRISMA).^27^

### Search Strategy

The search strategy was based on patient, intervention, comparison, and outcome (PICO).^15^ A structured PICO question was developed, where P stood for human or animal cells and tissues, I for the exposition to residual monomers contained in polymers used in dentistry, C for unexposed cells or tissues, and O for DNA damage or chromosomal aberrations. The full question read as follows: ‘Do any residual monomers contained in polymers used in dentistry generate DNA damage or chromosomal aberrations?’

### Databases and Data Collection Process

An electronic search was carried out in the databases MEDLINE, EBSCO, SCiELO, BVS LILACS, COCHRANE, and ScienceDirect, from January 1, 2000 to June 30, 2020, using the terms and their combinations: ‘Micronucleus’ OR ‘Genotoxicity’ OR ‘DNA damage’ AND ‘Dental Resin’ OR ‘Poly-Methyl Methacrylate’ OR ‘Residual Monomers’. The search was carried out in the English language using the MeSH terms described, with the help of Boolean operators (OR, AND) to combine the searches (Table 1).

**Table 1:** Databases and data collection process

Databases	Records Identified
MEDLINE	366
EBSCO	115
SCiELO	1
COCHRANE	1
BVS LILACS	14
SCIENCE DIRECT	285
Total	782
	

### Inclusion and Exclusion Criteria

The search was carried out in the databases of the last 20 years of publications, including in vitro studies in human or animal cells, clinical trials in humans or animals that measured or evaluated the genotoxic potential of some of the residual monomers and restorative materials used in dentistry. The exclusion criteria were case reports, review articles, studies, or trials without a measurement of genetic damage or chromosomal alterations, polymers or monomers used outside the dental area, and studies or trials without control groups.

### Selection of Studies

The titles and summaries of the reports identified in the searches were read independently by two authors. For the studies that met the inclusion criteria or whose title and abstract information were not sufficient, a complete reading of the study was performed for decision making. Disagreements were discussed and resolved between the two authors. In case of not reaching an agreement, the analysis of a third reviewer was considered. Finally, the approved articles were contemplated for inclusion in this study. Aspects such as cell lineage, type of monomer or co-monomer, and genotoxicity assay were treated in a manner analogous to the PICO components of a trial.^15^

### Quality Evaluation

This study was limited by the absence in the published literature of randomised clinical trials evaluating the genotoxicity of residual monomers used in restorative interventions, the lack of specific validated guidelines for systematic reviews of nonclinical studies, and the lack of external validity among in vitro studies.^39^ To overcome these limitations and promote quality and transparency of evidence among the in vitro studies, the Checklist for Reporting In Vitro Study Guidelines was used to evaluate each study according to the article’s description of the following five parameters for study quality assessment: description of sample size calculation (sample size calculation was one of the steps in methodology), description of the sample preparation and handling (detailed explanation of sample preparation and handling, and information on sample loss), randomisation and blinding (two or more independent observers or researchers, allocating samples, and maintaining a certain degree of blinding of samples), statistical analysis (use of the appropriate statistical method for analysing data), and meaningful differences between groups (measure that would make a difference clinically or scientifically).^22^ If the author reported the parameter the article received a ‘Y’ (yes); if it was not possible to find the information, the article received an ‘N’ (no). Articles that reported one or two parameters were classified as having a high risk of bias, three to four items as medium risk of bias, and five items as low risk of bias.

## Results

**Fig 1:**
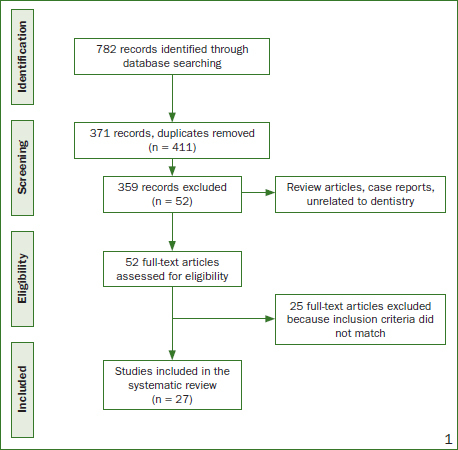
Study flowchart.

A total of 782 publications were located, and 371 duplicate articles were identified. Of the remaining 411, titles and abstracts were analysed regarding the objective of the study and its relevance to dentistry. Three hundred fifty-nine were eliminated, and 52 potentially relevant articles were selected and downloaded for complete reading and analysis by the two authors. Twenty-seven studies were selected for analysis of this systematic review, according to the inclusion criteria (Fig 1). Results are shown in Table 2 for in vitro studies and in Table 3 for in vivo studies, to facilitate the distribution and visualisation of the data.

**Table 2:** In vitro reports included in this review showing genotoxic effects of monomers used in restorative dentistry

Assay	Groups	Results	Reference
Micronucleus test in hamster lung fibroblasts	HEMA at 0 mM at 4000 mM Bis-GMA at 0 mM at 75 mM UDMA at 0 mM at 75 mM TEG-DMA At 0 mM At 750 mM MMA At 0 mM At 2 x 10^4^ mM	4.3 MN/1000 cells 22.7 MN/1000 cells 8.3 MN/1000 cells 15.3 MN/1000 cells 12.7 MN/1000 cells 21 MN/1000 cells 9.3 MN/1000 cells 78.3 MN/1000 cells 7.3 MN/1000 cells 9.7 MN/1000 cells	Schweilk, 2001 Germany
DNA synthesis inhibition assay and sister-chromatic exchange (SCE) in hamster ovary cells	MMA: 9.33x10^-1^ mg/ml 9.33x10^-2^ mg/ml 9.33x10^-3^ mg/ml 9.33x10^-4^ mg/ml Negative control: untreated cells	90% inhibition – 6.70 SCE/cell* 80% inhibition – 8.33 SCE/cell* 63% inhibition* – 7.63 SCE/cell* 55% inhibition* – 8.25 SCE/cell* 100% inhibition – 4.23 SCE/cell	Yang, 2003 Taiwan
Comet Assay in human peripheral lymphocytes	HEMA at 2.5x10^-2^ M Bis-GMA at 2.5x10^-2^ M UDMA at 2.5x10^-2^ M TEG-DMA at 2.5x10^-2^ M Negative control DMSO	OTM 3.3 * OTM 7.4 * OTM 2.82 * OTM 4.5 * OTM 1.0-1.2	Kleinsasser, 2004 Germany
Comet Assay in human lymphocytes and parotid gland tissue	HEMA at 2.5 x 10^-2^ M TEG-DMA at 2.5 x 10^-2^ M UDMA at 2.5 x 10^-2^ M Negative Control DMSO	Parotid: OTM 9.7 * Lymphocytes: OTM 6.1 * Parotid: OTM 10.7 * Lymphocytes: OTM 8.8 * Parotid: OTM 10.5 * Lymphocytes: OTM 6.4 * OTM 2.2	Kleinsasser, 2006 Germany
Micronucleus test in hamster fibroblasts	Clearfil SE Bond (Kuraray) Clearfil Protect Bond (Kuraray) AdheSE (Ivoclar Vivadent) Prompt L-Pop (3M Oral Care) Excite (Ivoclar Vivadent) Negative control: ethanol	>10 MN/ 1000 cells * ≤10 MN/1000 cells >10 MN/1000 cells * >10 MN/1000 cells * >10 MN/1000 cells 10 MN/1000 cells	Demirci, 2008 Germany
Sister-chromatic exchange (SCE) in human lymphocytes	Hibrid composites: Tetric Ceram (Ivoclar Vivadent) Filtek 250 (3M Oral Care) Nanohibrid composite: Simile (Pentron) Laboratory composite: Adoro (Ivoclar Vivadent) Negative control DMSO	19.79 SCE/cell * 15.10 SCE/cell * 13.15 SCE/cell 11.45 SCE/cell 10.79 SCE/cell	Bakopoulou, 2008 Greece
Fluorimetric Detection of Alkaline DNA Unwinding (FADU) in human gingival fibroblasts	HEMA at 2.5 mM Bis-GMA at 0.25 mM MMA at 2.5 mM TEG-DMA at 2.5 mM Negative control: untreated cells	>60% DNA integrity 25% DNA integrity * >60% DNA integrity >60% DNA integrity 68% DNA integrity	Durner, 2010 Germany
Comet Assay in human peripheral lymphocytes	HEMA at 2.5 mM HEMA at 5 mM HEMA at 10 mM Negative control: unexposed cells	>60% tail DNA (DNA damage) >80% tail DNA (DNA damage) * 100% tail DNA (DNA damage) * <60% tail DNA (DNA damage)	Pawlowska, 2010 Poland
Comet Assay in human peripheral lymphocytes	UDMA at 0.1 mM UDMA at 0.25 mM UDMA at 0.5 mM UDMA at 0.75 mM UDMA at 1 mM Negative control: unexposed cells	<10% tail DNA (DNA damage) * >10% tail DNA (DNA damage) * >10% tail DNA (DNA damage) * 20% tail DNA (DNA damage) * >30% tail DNA (DNA damage) * <5% tail DNA (DNA damage)	Poplawski, 2010 Poland
H2AX- Inmunofluorescence in human gingival fibroblasts	HEMA at 11.2 mM Bis-GMA at 0.09 mM UDMA at 0.1 mM TEG-DMA at 3.6 mM Negative control DMSO	>2 foci/cell * 5 foci/cell * 3 foci/cell * >2 foci/cell * <1 foci/cell	Ucran, 2010 Germany
Comet Assay in human gingival fibroblasts	Monomer mixture bis-GMA/TEG-DMA (55/45) At 0.05 mM At 0.10 mM At 0.20 mM Negative control: unexposed cell	>20% tail DNA (DNA damage) * 30% tail DNA (DNA damage) * <40% tail DNA (DNA damage) * <5% tail DNA (DNA damage	Blasiak, 2012 Poland
Comet Assay in human leukocyte cells	Elutes of freshly cured: Tetric Evoceram (Ivoclar Vivadent) Tetric Evoflow (Ivoclar Vivadent) Gradia Direct Posterior (GC) Gradia Direct flow (GC) Filtek z250 (3M Oral Care Filtek Supreme XT flow (3M Oral Care) Negative control: Saline solution	1.5% tail DNA (DNA damage) * >1.8% tail DNA (DNA damage) * 1.8% tail DNA (DNA damage) * 1.5% tail DNA (DNA damage) * >0.8% tail DNA (DNA damage) 0.6% tail DNA (DNA damage) 0.8 tail DNA (DNA damage)	Tadin, 2013 Croatia
H2AX- Inmunofluorescence in human gingival fibroblasts	Bis-GMA at 90 µM Bis-GMA at 30 µM Bis-GMA negative control UDMA at 100 µM UDMA at 33.5 µM UDMA negative control GMA at 2500 µM GMA negative control	4.05 foci/cell * 2.12 foci/cell * 1.39 foci/cell 2.5 foci/cell * 2.21 foci/cell * 1.39 foci/cell 2.57 foci/cell * 1.39 foci/cell	Lottner, 2013 Germany
Comet Assay in human gingival and pulp fibroblasts	Versatile flow, Kerr (self-adhering flowable composite) Kalore, GC (nano-hybrid resin composite) Negative control: medium only	(Gingival) 2.5 tail intensity (% DNA) * (Pulp) 1.6 tail intensity (% DNA) * (Gingival) 1.2 tail intensity (% DNA) (Pulp) 4.0 tail intensity (% DNA) * (Gingival) 1.1 tail intensity (% DNA) (Pulp) 0.4 – 0.6 tail intensity (% DNA)	Tadin, 2014 Croatia
Comet Assay and micronucleus test in human lymphocytes	HEMA at 10 µM HEMA at 100 µM HEMA at 1 mM TEG-DMA at 1 µM TEG-DMA at 10 µM TEG-DMA at 100 µM Negative control: DMSO	OTM 0.4 and 1.3 M/N 1000 cells OTM 0.5 and 1.4 M/N 1000 cells OTM 1.5 * and 2.5 M/N 1000 cells OTM 0.4 and 1.4 M/N 1000 cells OTM 0.5 and 1.5 M/N 1000 cells OTM 1.2 * and 1.4 M/N 1000 cells OTM 0.5 and 1.1-1.2 M/N 1000 cells	Ginzkey, 2015 Germany
H2AX/53BP1 focus assay in human gingival fibroblasts	Bis-GMA: 0.012 mM 0.03 mM 0.1 mM 0.3 mM GMA: 0.0036 mM 0.009 mM 0.03 mM 0.09 mM Negative control DMSO	.7 av. Foci/cell * 2.2 av. Foci/cell * 2.8 av. Foci/cell * 3.8 av. Foci/cell * 0.7 av. Foci/cell * 0.9 av. Foci/cell * 1.9 av. Foci/cell * 2.7 av. Foci/cell * 0.4 av. Foci/cell	Styllou, 2015 Germany
Comet Assay and micronucleus test in human lymphocytes	Bulk-fill composite SDR (Dentsply) Bulk-fill composite Venus (Heraeus Kulzer) Bulk-fill composite X-tra Base (Voco) Conventional composite Tetric Evoflow (Ivoclar) Negative control: untreated cells	2.6 (% of DNA in tail) 7 MN/1000 cells 2.8 (% of DNA in tail) 7 MN/1000 cells 2.9 (% of DNA in tail) 7 MN/1000 cells 1.8 (% of DNA in tail) 10 MN/1000 cells 1.4 (% of DNA in tail) 6.5 MN/1000 cells	Taubock, 2016 Switzerland
H2AX- Inmunofluorescence in human gingival fibroblasts	Micro-hybrid composite Esthet.X (Dentsply) Micro-hybrid composite Venus (Heraeus Kulzer) Mulit-hybrid composite X-tra Fil (Voco) Micro-hybrid composite Clearfil AP-X(Kuraray) Nano-hybrid ormocer Admira (Voco) Micro-hybrid QuiXfil (Dentsply) Negative control: médium	0.43 foci/cell * 0.39 foci/cell * 0.26 foci/cell 0.28 foci/cell 0.20 foci/cell 0.23 foci/cell 0.22 foci/cell	Yang, 2018 Germany
Comet Assay in mouse fibroblasts	Self-adhesive resin cement G-Cem (GC). UDMA and TEG-DMA Self-adhesive resin cement Speed-Cem (Ivoclar). UDMA and TEG-DMA Self-adhesive resin cement Relyx U200 (3M Oral Care) UDMA and TEG-DMA Negative control DMSO	250 tail intensity (% control) 200 tail intensity (% control) * 340 tail intensity (% control) * 100 tail intensity (% control)	Kurt, 2018 Turkey
Micronucleus assay in human lymphocytes	Eluate of Tetric EvoCeram (Ivoclar) At 0.007 g/ml after 4 h At 0.013 g/ml after 4 h At 0.007 g/ml after 24 h At 0.013 g/ml after 24 h Eluate of Tetric EvoFlow (Ivoclar) At 0.007 g/ml after 4 h At 0.013 g/ml after 4 h At 0.007 g/ml after 24 h At 0.013 g/ml after 24 h Eluate of Filtek Ultimate (3M Oral Care) At 0.007 g/ml after 4 h At 0.013 g/ml after 4 h At 0.007 g/ml after 24 h At 0.013 g/ml after 24 h Eluate of Filtek Ulimtate Flow (3M Oral Care) At 0.007 g/ml after 4 h At 0.013 g/ml after 4 h At 0.007 g/ml after 24 h At 0.013 g/ml after 24 h Eluate of G-aenial (GC) At 0.007 g/ml after 4 h At 0.013 g/ml after 4 h At 0.007 g/ml after 24 h At 0.013 g/ml after 24 h Eluate of G-aenial Flo (GC) At 0.007 g/ml after 4 h At 0.013 g/ml after 4 h At 0.007 g/ml after 24 h At 0.013 g/ml after 24 h Negative control: exposed only to medium	7 MN/1000 cells 10 MN/1000 cells 5 MN/1000 cells 8 MN/1000 cells 5 MN/1000 cells 6 MN/1000 cells 9 MN/1000 cells 4 MN/1000 cells 3 MN/1000 cells 2 MN/1000 cells 2 MN/1000 cells 4 MN/1000 cells 1 MN/1000 cells 2 MN/1000 cells 5 MN/1000 cells 5 MN/1000 cells 3 MN/1000 cells 4 MN/1000 cells 4 MN/1000 cells 4 MN/1000 cells 2 MN/1000 cells 4 MN/1000 cells 4 MN/1000 cells 3 MN/1000 cells 2 MN/1000 cells	Brzovic, 2018 Croatia

*Statistically significant compared to control (p<0.05).

**Table 3:** In vivo reports included in this review showing genotoxic effects of monomers used in restorative dentistry

Assay	Groups	Results	Reference
Comet Assay in human lymphocytes	40 subjects carrying dental fillings (20 males, 24 females) Control group: 24 individuals (13 males, 11 females)	OTM 65. 8 * 42.1 tail intensity (% tail DNA) * OTM 35.4 28.5 tail intensity (% tail DNA)	Dipietro, 2008 Italy
Micronucleus test in Wistar rat erythrocytes	16 rats exposed to MMA vapor for 8 h 8 rats receiving cyclophosphamide (positive control) 8 rats receiving water and food ad libitum (negative control)	7 MN/1000 cells after 24 h * 2 MN/1000 cells after 5 days. 9 MN/1000 cells * 0.75 MN/1000 cells	Araujo, 2012 Brazil
Micronucleus assay in human lymphocytes	54 male dental technicians Control group: 38 male clerical workers, not exposed to metal alloys or other chemicals during work or leisure time	8.5 MN/1000 cells * 4.1 MN/1000 cells	Ishikawa, 2012 Japan
Micronuleus test and Comet Assay in human exfoliative cells from oral mucosa	43 subjects with restorative fillings (males and females, age interval of 18-28) Control group: 20 subjects with no restorative fillings (males and females, age interval of 18-28)	0.25% MN/1000 cells * >80% of DNA in the tail (TDNA) * 0.12% MN/1000 cells 60% of DNA in the tail (TDNA)	Visalli, 2012 Italy
Micronucleus test in human bucal mucosal cells	13 dental technicians working in a prosthetic production laboratory for at least 1 year Control group. 14 students and doctors	5.21 MN/1000 cells 6.23 MN/1000 cells	Azhar, 2013 Kindom of Saudi Arabia
Micronucleus test and Comet assay in human gingival epithelial cells	15 patients (38-59 years of age) receiving restorations with nanohybrid composite Kalore (GC) 15 patients (38-59 years of age) receiving restorations with self-adhering composite Vertise Flow (Kerr) Negative control: immediately before restoration	7 days: 4% tail DNA* 7 MN/1000 cells * 30 days: 5% tail DNA* 6.5 MN/1000 cells * 180 days: 5% tail DNA* 4.5 MN/1000 cells 7 days: 4% tail DNA* 6 MN/1000 cells * 30 days: 5% tail DNA* 6 MN/1000 cells * 180 days: 5% tail DNA* 4 MN/1000 cells 0 days: 2% tail DNA 4 MN/1000 cells	Tadin, 2013 Croatia
Micronucleus assay in human exfoliated epithelial cells from oral mucosa	26 Dental surgeons Control group: 26 individuals not related to the profession 19 dental technicians Control group: 19 individuals not related to the profession	1.6 MN/1000 cells * 0.6 MN/1000 cells 1.7 MN/1000 cells * 0.7 MN/1000 cells	Molina, 2019 Mexico

* Statistically significant compared to control (p<0.05).

### Assessment of Risk of Bias

Of the 27 studies included in this work, six studies presented a high risk of bias and 21 studies showed a medium risk of bias. None of the studies possessed all five parameters to be considered as having a low risk of bias. The results are described in Tables 4 and 5 according to the parameters considered in the analyses.

**Table 4:** Quality of evidence and assessment of risk of bias

	Sample size calculation	Sample preparation and handling	Randomisation and blinding	Statistical analysis	Meaningful differences between groups	Risk of bias
Schweilk, 2001	N	Y	N	N	Y	High
Yang, 2003	N	Y	N	Y	Y	Medium
Kleinsasser, 2004	N	Y	N	Y	Y	Medium
Kleinsasser, 2006	N	Y	N	Y	Y	Medium
Demirci, 2008	N	Y	Y	Y	Y	Medium
Bakopoulou, 2008	N	Y	Y	Y	Y	Medium
Durner, 2010	N	Y	N	Y	N	High
Pawloska, 2010	N	Y	N	Y	Y	Medium
Poplawski, 2010	N	Y	N	Y	Y	Medium
Ucran, 2010	N	Y	N	Y	Y	Medium
Blasiak, 2012	N	Y	N	Y	Y	Medium
Tadin, 2013	N	Y	N	Y	Y	Medium
Lottner, 2013	N	Y	N	Y	Y	Medium
Tadin, 2014	N	Y	N	Y	Y	Medium
Ginzkey, 2015	N	Y	N	Y	N	High
Styllou, 2015	N	Y	N	Y	Y	Medium
Taubock, 2016	N	Y	N	Y	N	High
Yang, 2018	N	Y	N	Y	Y	Medium
Kurt, 2018	N	Y	N	Y	Y	Medium
Brzovic, 2018	N	Y	N	Y	N	High
Dipietro, 2008	N	Y	Y	Y	Y	Medium
Araujo, 2012	N	Y	Y	Y	Y	Medium
Ishikawa, 2012	N	Y	N	Y	Y	Medium
Visalli, 2012	N	Y	Y	Y	Y	Medium
Azhar, 2013	N	Y	N	Y	N	High
Tadin, 2013	N	Y	Y	Y	Y	Medium
Molina, 2019	N	Y	N	Y	Y	Medium

**Table 5:** Risk of bias results of studies included in this review

Risk of bias	Number of reports
Low risk of bias	0
Medium risk of bias	21
High risk of bias	6
Total number of reports	27

## Discussion

From the initial electronic research, 27 studies fulfilled all the required criteria for eligibility. Using these, two categories were constructed based on the experimental design, in vivo and in vitro reports. As shown in Table 2 for the in vitro research, two main methods of assessing DNA damage were reported: micronucleus (MN) counting and alkaline comet assay.^4,5,6,8,10,12,14,18,19,22,23,32,34,36,38,40,41,42,44,47,48^ As shown in Table 3 for in vivo reports, the main method for assessing genotoxic damage was MN counting.^1,3,11,17,28,39,46^

The MN assay is a mutagenic test system that is frequently used in in vitro and in vivo toxicological screening for detecting potential genotoxic compounds that lead to the induction of small DNA fragments (micronuclei) in the cytoplasm of the dividing cells. Micronuclei can be observed as chromosome fragments produced by DNA strand breakage, or as whole chromosomes that have been formed during the anaphase of mitosis or meiosis when they were not able to migrate with the rest of the chromosomes.^6^ The Comet assay is a microgel technique involving electrophoresis under alkaline (pH >13) conditions for detecting DNA damage in single cells. At this pH, increased DNA migration is associated with raised levels of frank single-strand breaks (SSBs). Because genotoxic agents induce different magnitudes of SSB, this assay provides great sensitivity for identifying genotoxic agents.^10^ Irrespective of the method, however, the analysis of DNA damage performed in most of the studies included in this systematic review (23 out of 27) (Tables 2 and 3) revealed alterations in DNA stability by MN assay, comet assay, sister-chromatic exchange, and immunofluorescence.

In the in vitro studies, the cells most commonly used to show a genotoxic effect with exposure to monomers were peripheral lymphocytes (9 of 20) and gingival fibroblasts (7 of 20) (Table 2). The collection of buccal cells is arguably the least invasive method available for measuring DNA damage in humans, especially in comparison to obtaining blood samples for lymphocyte and erythrocyte assays, or tissue biopsies.^43^ Because the clinical use of these dental materials involves direct contact with oral tissues, the information collected from in vitro studies using gingival cells can be considered more accurate and less invasive in terms of analysing a local genotoxic effect. Only one of these studies performed on gingival cells did not show an association between the use of composites and genotoxicity measured with the MN assay.^8^

Several reviews are available on the application of the MN assay in exfoliated cells,^16,24,35^ showing the usefulness of the MN test applied in buccal cells to assess the genotoxic impact of environmental and occupational exposure, lifestyle, and malnutrition in intervention studies. Moreover, the strong correlation of MN frequency in exfoliated buccal cells with MN frequency in lymphocytes implies that systemic genotoxic effects within the bloodstream may also impact on and be detectable in buccal cells. One of the main limitations of the assay, which needs to be addressed in the context of practical application, is the large variability in MN frequency observed across laboratories, patients, and control groups. Nevertheless, our review has some limitations, such as heterogeneity in the study design, with different schemes of subject recruitment, and the use of different experimental protocols.^6^

Through electronic research, it is notable that the most common monomers/co-monomers associated with increased levels of reactive oxygen species (ROS) and DNA damage (genotoxic effect) are bis-GMA, HEMA, TEG-DMA, and UDMA (Tables 2 and 3), which are used as bonding resins and direct restoration materials, and are present in some cements, dentin adhesives, and sealing agents, as well as in bonding of orthodontic brackets. Several studies have shown that these monomers and other components were released from restorative materials into the oral environment either from incomplete polymerisation or because of resin degradation. This degradation can occur through a variety of physical and chemical mechanisms, such as dissolution and disintegration in saliva, mechanical wear through chewing forces, bacterial activity and erosion by food.^2,11,25,26,49^

Nevertheless, results regarding an association with a genotoxic effect from in vitro studies should be viewed with caution, due to an absence of correlation between the concentrations used in in vitro studies and actual clinical situations. How high is the concentration of monomers leached from a class-I cavity restored with composite resin? Some studies mention that the surface area of the sample used (220 mm^2^) is four times larger than that of typical restorations (52 mm^2^).^48^ It has been demonstrated that a larger surface area of the sample increases the release of components. Furthermore, the presence of an oxygen inhibition layer also contributes to an increased number of released components. However, in a clinical situation, the exposed surface area is limited, and the oxygen inhibition layer will be removed by grinding and polishing.^48^ Other authors mention that composites eluted into 75% ethanol/25% water solution should be no cause for alarm for several reasons: first, normal alcoholic drinks have lower concentrations of ethanol. Second, the contact time between ethanol, the composite, and then the oral cells is exceptionally low; therefore, the elution time is much shorter than 24 h as used in experiments.^12^

In most in vivo studies (4 of 7), the samples were taken from the oral mucosa and the DNA damage was registered with the MN test. Some of these reports even use both methods, MN assay and comet assay, at the same time.^11,39,46^ The combined comet/MN assay protocol has proven to be a sensitive and effective method for detecting multiple classes of genotoxins across a wide range of target organs within the same patient.^45^ As for studies that analysed patients with resin restorations (3 of 7), a genotoxic effect was demonstrated by comparing the measurements obtained from the group with resin restorations and their negative controls using the MN and the Comet assay.^11,39,46^

Of the studies that evaluated a genotoxic effect caused by occupational exposure to monomers, only one did not report a genotoxic effect measured with the MN test. However, this type of exposure entails a series of larger variables in the study subjects, such as workplace ventilation, the type and use of face masks, the use of gloves, and the use of protective glasses.^1,3,17,28^

For in vivo studies, it may be a limitation that the oral cavity is a multifactorial environment and that each patient bears his/her specific biological variation. Several biological, ecological, demographic, and lifestyle factors can influence in vivo analyses, which are therefore difficult to standardise.^7,43^

It must also be mentioned that data revealing the potential genotoxic effect are based upon dental material, composites, and adhesives used in each study. New dental materials, composites, self-adhesive composites and techniques are constantly introduced in the field of restorative dentistry, in which monomer leaching and monomer elution may differ from the materials analysed in this study. Data regarding a possible genotoxic effect of the most recent materials are not yet available.^29,30,31^

## Conclusion

From the electronic search, structured data extraction, and analysis by different independent reviewers, results of this systematic review allow us to conclude that DNA damage is induced by monomers/co-monomers (TEG-DMA, bis-GMA, UDMA, and HEMA) used in restorative dentistry.

However, this finding should be interpreted with caution. The concentrations used in in vitro studies are heterogeneous; therefore, making comparisons between them is difficult. Furthermore, the general lack of correlation between the dosage of leachable monomers used for experiments and actual clinical situations is illustrated. As for the validation of existing in vivo studies, large-scale prospective studies in patients with dental restorations are still required, since evidence from available research is still insufficient. Further efforts are needed to carry out controlled randomised clinical trials, which are urgently required to explore the extent to which genotoxicity can be observed for these materials in clinical situations.
